# Salivary *Prevotella* qPCR Signal as an Exploratory Non-Invasive Adjunct for Rotterdam Phenotype Stratification in Women with Polycystic Ovary Syndrome: A Proof-of-Concept Cross-Sectional Study

**DOI:** 10.3390/diagnostics16132041

**Published:** 2026-06-30

**Authors:** Arif Tunjungseto, Oni As’ad Hadi, Intan Winta Pratiwi, Fakhriyah Iffatunnisa, Fadhil Ahsan, Budi Santoso

**Affiliations:** 1 Doctoral Program of Medical Science, Faculty of Medicine, Universitas Airlangga, Surabaya 60132, Indonesia; arif.tunjungseto@fk.unair.ac.id; 2 Department of Obstetrics and Gynecology, Faculty of Medicine, Universitas Airlangga, Surabaya 60132, Indonesia; intanwintap@gmail.com (I.W.P.); fakhriyahiffatunnisa@gmail.com (F.I.); 3 Medical Program, Faculty of Medicine, Universitas Airlangga, Surabaya 60132, Indonesia; onihadiwork@gmail.com; 4 Department of Obstetrics and Gynecology, University Hospital of Würzburg, 97080 Würzburg, Germany; fadhilahsan@gmail.com

**Keywords:** polycystic ovary syndrome (PCOS), salivary microbiota, *Prevotella*, qPCR, Cq value, non-invasive diagnostics, diagnostic adjunct, Rotterdam phenotype, phenotype stratification, proof-of-concept

## Abstract

**Background/Objectives**: Polycystic ovary syndrome (PCOS) is a heterogeneous endocrine-metabolic disorder. Rotterdam-defined phenotypes may need adjunctive, non-invasive signals for research-level phenotypic stratification. Saliva deserves attention because it is easy to collect repeatedly, acceptable in outpatient settings, and may reflect oral-systemic inflammatory and endocrine-metabolic interactions. This study evaluated whether salivary qPCR signals for *Lactobacillus*, *Prevotella*, and *Bifidobacterium* differ across Rotterdam-defined PCOS phenotypes and controls, with emphasis on the exploratory relevance of *Prevotella*. **Methods**: This cross-sectional proof-of-concept study included 110 women: 87 with PCOS and 23 controls. PCOS phenotypes were classified according to the Rotterdam criteria. Salivary microbial targets were assessed using SYBR Green-based genus-specific qPCR. Available instrument-export spreadsheets were reviewed for standard-curve quality control. Because the available assay outputs did not support robust absolute quantification, inferential analyses used Cq-based microbial signals only. Lower Cq values indicate stronger target DNA signal. No universal bacterial 16S rRNA reference, exogenous spike-in, salivary flow correction, or fully validated copy-number conversion was available. Group differences were evaluated using non-parametric tests with Bonferroni-corrected post-hoc comparisons. ROC, regression, and correlation analyses were retained as hypothesis-generating analyses only. **Results**: *Prevotella* Cq values differed significantly across groups (*p* < 0.001), with lower median Cq values in phenotypes A, B, and C than in phenotype D and controls. *Lactobacillus* Cq values did not differ significantly (*p* = 0.249). *Bifidobacterium* showed an overall group difference (*p* < 0.001), but its pattern and assay performance were less consistent. Among women with PCOS, *Prevotella* Cq values were associated with Ferriman-Gallwey score and polycystic ovarian morphology. **Conclusions**: Salivary *Prevotella* showed the clearest exploratory genus-level qPCR signal across Rotterdam-defined PCOS phenotypes. The findings support further technical and clinical validation of saliva-based microbial profiling as a possible adjunct to conventional PCOS phenotyping. They do not validate *Prevotella* as a standalone diagnostic biomarker, do not define clinical cutoffs, and do not quantify absolute bacterial load.

## 1. Introduction

Polycystic ovary syndrome (PCOS) is a common endocrine disorder among reproductive-age women and is characterized by heterogeneous reproductive, endocrine, and metabolic manifestations. The Rotterdam criteria remain widely used for clinical classification and generate four major phenotypes based on combinations of hyperandrogenism, ovulatory dysfunction, and polycystic ovarian morphology [1,2]. This phenotypic heterogeneity is clinically important because different phenotypes may carry different reproductive, metabolic, and inflammatory implications [3,4,5,6,7,8].

Microbiome research has become increasingly relevant to PCOS because microbial ecosystems may interact with androgen exposure, insulin resistance, low-grade inflammation, bile acid metabolism, and immune regulation [9,10,11,12,13,14,15,16]. Most PCOS microbiome studies have focused on the gut or vaginal microbiome [10,17,18,19,20,21,22,23,24,25,26,27,28,29,30,31]. Vaginal microbiome assessment is scientifically important and can be performed using non-invasive or minimally invasive sampling, but it represents a local reproductive-tract niche. Saliva addresses a different question. It captures oral microbial ecology and may provide a repeatable outpatient matrix for studying oral-systemic signals related to endocrine-metabolic disease.

Saliva specifically deserves evaluation in PCOS for three reasons. First, it can be collected repeatedly without pelvic sampling, which may improve acceptability for screening-oriented or longitudinal studies. Second, salivary microbiome studies in PCOS have reported altered oral microbial community features and disease-related associations, although the evidence remains limited and heterogeneous [9,32]. Third, oral and gut microbiota have been discussed in several female reproductive and gynecological disease contexts, including gynecological cancers, through immune, inflammatory, and hormone-related pathways [33]. These data do not prove diagnostic value in PCOS, but they justify saliva as a distinct exploratory matrix rather than a substitute for vaginal or gut microbiome profiling.

Among candidate genera, *Lactobacillus*, *Bifidobacterium*, and *Prevotella* are biologically plausible exploratory targets because prior studies, mainly in intestinal ecosystems, link these genera to barrier function, host metabolic regulation, dietary patterns, immune signaling, and inflammatory tone [34,35,36,37,38,39,40]. *Prevotella* is especially context-dependent: different species and strains may be associated with either eubiotic fiber-related profiles or dysbiotic inflammatory states depending on host and environmental conditions [37,38,39,40]. Genus-level qPCR therefore cannot identify whether a detected *Prevotella* signal reflects beneficial, commensal, or potentially pathogenic species.

Accordingly, this study analyzed qPCR-derived salivary Cq signals of *Lactobacillus*, *Prevotella*, and *Bifidobacterium* across Rotterdam-defined PCOS phenotypes and controls. The objective was not to replace hormonal assessment, ultrasound, clinical phenotyping, or vaginal microbiome assessment. The objective was to examine whether genus-specific salivary microbial signals, particularly *Prevotella*, show phenotype-stratified patterns that could justify future validation as a non-invasive adjunct to conventional PCOS phenotype assessment.

## 2. Materials and Methods

This study used a cross-sectional design. Participants were recruited from premenopausal and infertile women who regularly attended outpatient clinical services. PCOS was diagnosed according to the Rotterdam criteria. Phenotype A was defined as hyperandrogenism, ovulatory dysfunction, and polycystic ovarian morphology; phenotype B as hyperandrogenism and ovulatory dysfunction without polycystic ovarian morphology; phenotype C as hyperandrogenism and polycystic ovarian morphology without ovulatory dysfunction; and phenotype D as ovulatory dysfunction and polycystic ovarian morphology without hyperandrogenism [1,2]. Controls were women without clinical PCOS according to the same diagnostic framework.

Eligible participants were women aged 18–42 years, clinically diagnosed with PCOS according to the Rotterdam criteria or classified as controls, willing to participate in the study, able to provide written informed consent, and willing to undergo the required research procedures, including blood and saliva sampling. Participants with incomplete key clinical or salivary qPCR data were excluded from the final analysis.

The final analysis set included 110 women in total, comprising 87 women with PCOS and 23 controls. Baseline variables recorded for analysis included age, Ferriman-Gallwey score, menstrual irregularity or ovulatory dysfunction, and polycystic ovarian morphology on transvaginal ultrasonography. Ferriman-Gallwey scoring was used as the clinical measure of hirsutism [41]. Complete biochemical androgen, gonadotropin, anti-Mullerian hormone, fasting insulin, HOMA-IR, BMI, and periodontal datasets were not uniformly available in the analyzable dataset and therefore could not be incorporated into the main adjusted models.

Saliva was selected as an exploratory oral-systemic matrix, not as a replacement for vaginal microbiome sampling. Vaginal swabs were not collected because the study protocol focused on salivary qPCR targets and outpatient saliva sampling. Saliva samples were collected in the morning after 1–2 h of fasting. Participants were instructed not to brush their teeth, use mouthwash, or consume food immediately before sampling. Samples were collected into sterile containers and processed for DNA extraction. The sampling protocol did not include standardized salivary flow rate measurement, salivary pH, detailed periodontal examination, detailed oral hygiene scoring, exact dietary intake, or full adjustment for recent antibiotic or probiotic exposure. These missing variables are treated as major limitations.

Salivary microbial signals for *Lactobacillus*, *Prevotella*, and *Bifidobacterium* were assessed using genus-specific quantitative polymerase chain reaction (qPCR). DNA was extracted from saliva samples using the QIAamp DNA Stool Mini Kit (QIAGEN GmbH, Hilden, Germany). qPCR amplification was performed using GoTaq qPCR Master Mix (Promega Corporation, Madison, WI, USA) on a MyGo Mini S Real-Time PCR Cycler with MyGo Mini software version 3.5.6 (IT-IS Life Science Ltd., Mahon, Cork, Ireland). The reaction mixture consisted of 12.5 μL of master mix, 5 μL of nuclease-free water, 1 μL of forward primer, 1 μL of reverse primer, and 5.5 μL of DNA template, for a total reaction volume of 25 μL.

The thermal profile consisted of an initial denaturation at 95 °C for 120 s, followed by 40 amplification cycles of denaturation at 95 °C, target-specific annealing, and extension at 72 °C. Melting curve analysis was performed from 60 °C to 97 °C at 0.1 °C/s to assess amplification specificity. Instrument run files indicated an annealing temperature of 60 °C for *Lactobacillus* and *Prevotella* and 50 °C for *Bifidobacterium*. Genus-specific standard curves were generated using serially diluted standards ranging from 1.5 × 10^8^ to 1.2 × 10^9^ nominal units. For the present revision, the supplied instrument-export spreadsheets were reviewed to extract standard Cq values, calculated quantity outputs, slope, y-intercept, R2, amplification efficiency, duplicate standard Cq spread, and negative-control observations where available. Standard-curve QC was recalculated as the regression of Cq on log10 nominal standard concentration, and amplification efficiency was calculated as (10^(−1/slope)^ − 1) × 100. The exports did not contain full fluorescence amplification traces, melt-peak image outputs, extraction blank results, LOD/LOQ experiments, universal bacterial 16S normalization, spike-in recovery, or inter-run reproducibility data. Therefore, the available run files were sufficient only for transparent exploratory Cq-based reporting and partial standard-curve QC. They were not sufficient to establish validated absolute quantification, lower limit of detection, lower limit of quantification, inter-assay reproducibility, or clinical assay performance.

The inferential analysis was based on Cq values rather than GAPDH-normalized Delta Ct/Delta Delta Ct expression analysis. GAPDH was not used as a bacterial reference gene because it is a host housekeeping gene and is not an appropriate denominator for genus-level bacterial abundance in saliva. In addition, no universal bacterial 16S rRNA reference, exogenous spike-in control, salivary flow normalization, total DNA yield normalization, or fully validated absolute copy-number conversion was available for all targets. Broad-coverage bacterial 16S qPCR has been proposed as a way to quantify total bacterial load and can complement community-level microbiome data [42]. Therefore, the present results are reported only as qPCR-derived Cq signals. Lower Cq values indicate stronger target DNA signal. Standard-curve-derived quantity outputs and curve-quality parameters are presented only as supplementary quality-control information. No claim of absolute CFU/mL bacterial load is made. qPCR reporting was revised in accordance with current MIQE 2.0 principles of transparent assay description, validation, and cautious interpretation [43].

Normality was assessed using the Shapiro–Wilk test [44]. Because the data were not normally distributed, continuous variables are reported as median with minimum and maximum values. Between-group comparisons were performed using the Kruskal–Wallis test [45]. Pairwise post-hoc comparisons were performed using Mann–Whitney U tests with Bonferroni correction [46]. Correlation analyses among women with PCOS were performed using Spearman rank correlation. False discovery rate correction was applied to exploratory correlation analyses using the Benjamini-Hochberg procedure [47].

Additional exploratory analyses were performed to support interpretation of the observed *Prevotella*-related findings. ROC analyses and age-adjusted regression models were retained as supplementary hypothesis-generating analyses only. These analyses were not used to establish diagnostic thresholds or clinical cutoffs because the study was cross-sectional, single-center, underpowered for biomarker validation, and lacked external validation and full adjustment for oral-health, metabolic, hormonal, dietary, and medication-related confounders.

The study protocol was reviewed and approved by the Ethics Committee of the Faculty of Medicine, Universitas Airlangga, Surabaya, Indonesia. Written informed consent was obtained from all participants before data and sample collection.

## 3. Results

### 3.1. Baseline Characteristics

A total of 110 participants were included: 23 women in phenotype A, 19 in phenotype B, 22 in phenotype C, 23 in phenotype D, and 23 controls. Baseline characteristics are summarized in Table 1.

Age did not differ significantly across groups (*p* = 0.811). Ferriman-Gallwey score differed markedly across groups (*p* < 0.001), as expected from phenotype definitions. Menstrual irregularity or ovulatory dysfunction and polycystic ovarian morphology on TVS also differed significantly across groups. In the corrected dataset, all participants in phenotype A had polycystic ovarian morphology on TVS, consistent with the Rotterdam phenotype A definition.

BMI, biochemical hormonal profiles, insulin-resistance markers, salivary flow rate, periodontal status, detailed diet, and recent antibiotic/probiotic exposure were not available as complete analyzable variables. Consequently, the reported group comparisons should be interpreted as unadjusted or minimally adjusted exploratory findings rather than confounder-controlled clinical biomarker estimates.

### 3.2. Salivary Microbial qPCR Signals Across PCOS Phenotypes and Controls

Salivary microbial Cq values across PCOS phenotypes and controls are shown in Table 2. These values should be read as qPCR-derived Cq signals; lower Cq values indicate higher target DNA signal.

*Lactobacillus* Cq values did not differ significantly across phenotypes and controls (*p* = 0.249). *Prevotella* Cq values differed significantly across groups (*p* < 0.001), with lower median Cq values in phenotypes A, B, and C than in phenotype D and controls. Because lower Cq values indicate stronger target DNA signal, these findings suggest a phenotype-associated *Prevotella* molecular signal in selected hyperandrogenic and/or PCOM-related PCOS phenotypes. This does not demonstrate absolute bacterial enrichment because total bacterial biomass and extraction efficiency were not normalized.

### 3.3. Post-Hoc Pairwise Comparisons and Exploratory Discriminatory Analyses

Post-hoc pairwise comparisons were performed for genera with significant overall between-group differences. Significant comparisons after Bonferroni correction are summarized in Table 3; the complete pairwise comparison table is provided in the Supplementary Materials.

For *Prevotella*, phenotype A, phenotype B, and phenotype C differed from phenotype D and/or controls after correction, reinforcing that the strongest phenotype-related salivary qPCR signal was observed for *Prevotella*. For *Bifidobacterium*, significant corrected differences were observed for phenotype A versus control and phenotype C versus control, but these findings were treated as secondary because the *Bifidobacterium* assay showed less robust available quality-control support than the *Prevotella* assay.

### 3.4. Exploratory Correlation Analysis Among Women with PCOS

Exploratory correlation analyses were performed among women with PCOS only (*n* = 87). The main findings are summarized in Table 4.

Ferriman-Gallwey score was not associated with *Lactobacillus* Cq values, but it correlated positively with *Prevotella* Cq values and negatively with *Bifidobacterium* Cq values. PCOM on TVS correlated negatively with *Prevotella* Cq values, indicating that PCOM was associated with a stronger *Prevotella* target DNA signal. Menstrual irregularity or ovulatory dysfunction was not significantly associated with any of the three microbial Cq signals after FDR correction. Because Cq direction is inverse to target DNA signal, all correlation directions should be interpreted cautiously and should not be read as causal mechanisms.

### 3.5. Standard-Curve Quality-Control Review of qPCR Instrument Exports

The supplied qPCR Excel exports were reviewed to address the remaining technical-QC comments. The review confirmed that standard-curve and Cq data could be summarized transparently, but it also confirmed that the available files did not support validated absolute bacterial quantification. Table 5 summarizes the standard-curve QC findings that were incorporated into the revised interpretation.

## 4. Discussion

This cross-sectional proof-of-concept study examined genus-specific salivary qPCR signals of *Lactobacillus*, *Prevotella*, and *Bifidobacterium* across Rotterdam-defined PCOS phenotypes and controls. The revised analysis deliberately reports Cq-based microbial signals rather than absolute bacterial abundance or GAPDH-normalized expression because the available assay files did not support uniformly robust absolute CFU/mL conversion. The additional review of the instrument-export spreadsheets improved technical transparency by adding standard-curve slopes, y-intercepts, R2 values, efficiency estimates, duplicate standard Cq spread, and available negative-control observations. However, these QC data reinforced rather than removed the need for cautious interpretation. Within this analytical frame, *Prevotella* showed the clearest and most interpretable phenotype-related pattern.

The main finding was that *Prevotella* Cq values differed significantly across PCOS phenotypes and controls. Phenotypes A, B, and C tended to show lower *Prevotella* Cq values than phenotype D and controls, indicating stronger *Prevotella* target DNA signal in selected PCOS phenotypes. This pattern is biologically plausible because these phenotypes include hyperandrogenism and/or PCOM-related features, which may reflect broader endocrine, inflammatory, and metabolic dysregulation [1,2,3,4,5,6,7,8,48,49,50]. However, the present data do not prove that *Prevotella* drives PCOS features, nor do they prove that PCOS causes salivary *Prevotella* changes.

The direction of the correlation findings requires careful interpretation. A positive correlation between Ferriman-Gallwey score and *Prevotella* Cq values means that higher hirsutism scores were associated with weaker *Prevotella* target signal, because higher Cq reflects lower target DNA signal. In contrast, the negative correlation between PCOM on TVS and *Prevotella* Cq values suggests that PCOM was associated with stronger *Prevotella* target DNA signal. These divergent associations reinforce the need to avoid simplistic claims that *Prevotella* is uniformly increased or decreased in PCOS.

*Prevotella* is biologically context-dependent. Depending on species, strain composition, diet, host metabolic status, periodontal ecology, and local mucosal conditions, *Prevotella* may be linked to fiber-rich dietary patterns, insulin sensitivity, or inflammatory dysbiosis [37,38,39,40]. Because the present study used genus-level qPCR rather than species-level sequencing, the data cannot determine whether the observed *Prevotella* signal reflects beneficial, commensal, or pathogenic *Prevotella* taxa. This is a key reason why the finding should be framed as an exploratory molecular signal rather than a mechanistic conclusion.

The *Bifidobacterium* results were statistically significant overall but less stable interpretively. Some corrected group differences were observed, particularly against controls. The supplied standard-curve export showed a positive slope (+1.766), very low R2 (0.058), and non-interpretable efficiency, indicating that the *Bifidobacterium* quantity output was not suitable for quantitative interpretation. Therefore, *Bifidobacterium* should be treated as a secondary exploratory finding requiring technical revalidation before biological or diagnostic interpretation.

A methodological strength of this study is the use of saliva as a non-invasive sample type, which is relevant for repeated outpatient research in reproductive endocrinology. Saliva was not chosen because it is superior to vaginal sampling. It was chosen because it addresses a different, more scalable oral-systemic diagnostic-adjunct question. Vaginal microbiome assessment remains important for local reproductive-tract ecology and should be included as a comparator in future studies. Another strength is explicit Rotterdam phenotype stratification rather than treating PCOS as a single homogeneous entity, because phenotype-level differences may be diluted when all PCOS cases are pooled.

Several limitations remain and should be read as central to the interpretation of this manuscript. First, the cross-sectional design prevents causal or temporal inference. Second, the microbiological analysis was genus-specific and qPCR-based, without 16S rRNA gene sequencing, shotgun metagenomics, species-level resolution, or functional profiling. Third, the newly incorporated qPCR spreadsheet review provided partial standard-curve QC, but the assays still did not meet the evidentiary standard required for validated absolute quantification. *Lactobacillus* showed low efficiency despite a moderate standard-curve fit, *Prevotella* had a discordant standard replicate and late negative-control amplification, and *Bifidobacterium* showed an uninterpretable standard curve. Fourth, the available files did not include complete amplification traces, melt-peak outputs, extraction blanks, LOD, LOQ, or inter-assay reproducibility for every target. Fifth, no universal bacterial 16S rRNA reference, exogenous spike-in, salivary flow correction, total DNA yield correction, or validated copy-number conversion was available. Sixth, BMI, insulin resistance, androgen biochemistry, diet, oral hygiene, periodontal status, recent antibiotic/probiotic exposure, medication use, and menstrual cycle phase were not fully included as covariates. Seventh, the study used a single-center sample with relatively small phenotype groups and lacked external validation. Eighth, exploratory ROC and regression analyses were not prespecified for clinical cutoff development and may be vulnerable to overfitting and type I error. Ninth, the salivary sampling protocol did not fully standardize diurnal variation or recent food intake beyond a 1–2 h fasting instruction.

Despite these limitations, the study provides a useful hypothesis-generating signal: salivary *Prevotella* Cq values differ across Rotterdam-defined PCOS phenotypes and show associations with selected diagnostic features. The findings justify larger, technically strengthened studies incorporating standardized oral examination, dietary and metabolic covariates, validated qPCR efficiency, universal bacterial reference targets or spike-in controls, absolute copy-number standards, species-level sequencing, vaginal and gut microbiome comparators, prespecified diagnostic thresholds, and independent validation cohorts.

## 5. Conclusions

In this cross-sectional proof-of-concept dataset, salivary *Prevotella* showed the clearest exploratory genus-level qPCR signal across Rotterdam-defined PCOS phenotypes. The revised interpretation is intentionally conservative: the study supports phenotype-associated variation in salivary microbial Cq signals as a candidate non-invasive adjunct for future research, but it does not establish *Prevotella* as a validated standalone diagnostic biomarker, does not quantify absolute bacterial load, and does not define clinical cutoffs. Future studies should combine saliva-based microbial profiling with robust qPCR validation, universal bacterial normalization or spike-in controls, species-level sequencing, metabolic and hormonal covariates, oral-health assessment, vaginal and gut microbiome comparators, prespecified diagnostic thresholds, and independent validation cohorts.

## Figures and Tables

**Table 1:** Baseline characteristics of study participants.

Characteristic	Phenotype A (*n* = 23)	Phenotype B (*n* = 19)	Phenotype C (*n* = 22)	Phenotype D (*n* = 23)	Control (*n* = 23)	*p*-Value
Sample size, *n*	23	19	22	23	23	-
Age, years	28 (19–40)	29 (22–38)	28 (18–42)	30 (21–40)	29 (20–38)	0.811
Ferriman-Gallwey score	8.0 (5.0–18.0)	7.0 (5.0–12.0)	6.5 (5.0–14.0)	3.0 (0.0–4.0)	0.0 (0.0–2.0)	<0.001
Menstrual irregularity or ovulatory dysfunction, *n* (%)	23 (100.0)	19 (100.0)	0 (0.0)	23 (100.0)	0 (0.0)	<0.001
Polycystic ovarian morphology on TVS, *n* (%)	23 (100.0)	0 (0.0)	22 (100.0)	23 (100.0)	0 (0.0)	<0.001

Continuous data are presented as median (minimum–maximum), while categorical data are presented as *n* (%). *p*-values for continuous variables were obtained using the Kruskal–Wallis test, and categorical variables were compared using appropriate exact/non-parametric procedures. TVS, transvaginal sonography.

**Table 2:** qPCR-derived salivary microbial Cq values across PCOS phenotypes and controls.

Microbiota	Phenotype A (*n* = 23)	Phenotype B (*n* = 19)	Phenotype C (*n* = 22)	Phenotype D (*n* = 23)	Control (*n* = 23)	*p*-Value
*Lactobacillus*	32.50 (12.34–36.82)	34.46 (27.84–37.22)	35.42 (11.76–36.98)	34.46 (26.64–37.00)	35.59 (27.74–36.85)	0.249
*Prevotella*	22.32 (19.11–35.68)	21.42 (18.70–35.95)	24.28 (18.21–32.20)	31.00 (19.44–37.03)	28.43 (20.54–36.77)	<0.001
*Bifidobacterium*	36.77 (27.94–37.25)	36.44 (11.76–37.16)	36.64 (35.35–37.09)	35.71 (31.96–37.13)	35.71 (5.92–37.01)	<0.001

Values are presented as median Cq (minimum–maximum). Lower Cq values indicate higher target DNA signal. *p*-values were obtained using the Kruskal–Wallis test. The values are not reported as absolute CFU/mL bacterial loads.

**Table 3:** Significant post-hoc pairwise comparisons after Bonferroni correction.

Microbiota	Comparison	Raw *p*-Value	Bonferroni-Adjusted *p*-Value	Interpretation
*Prevotella*	Phenotype A vs. Phenotype D	<0.001	0.010	Significant
*Prevotella*	Phenotype A vs. Control	<0.001	0.010	Significant
*Prevotella*	Phenotype B vs. Phenotype D	<0.001	0.010	Significant
*Prevotella*	Phenotype B vs. Control	<0.001	0.010	Significant
*Prevotella*	Phenotype C vs. Phenotype D	0.002	0.020	Significant
*Prevotella*	Phenotype C vs. Control	0.004	0.040	Significant
*Bifidobacterium*	Phenotype A vs. Control	<0.001	0.010	Significant
*Bifidobacterium*	Phenotype C vs. Control	<0.001	0.010	Significant

Pairwise comparisons were performed following the Kruskal–Wallis test. Only comparisons that remained significant after Bonferroni correction are shown. Complete post-hoc results are provided in Supplementary Table S1.

**Table 4:** Correlation between diagnostic PCOS features and salivary microbial Cq signals among women with PCOS.

Clinical Variable	Microbiota	*n*	Spearman rho	Raw *p*-Value	FDR-Adjusted *p*-Value	Interpretation
Ferriman–Gallwey score	*Lactobacillus*	87	0.040	0.712	0.937	Not significant
Ferriman–Gallwey score	*Prevotella*	87	0.423	<0.001	<0.001	Significant
Ferriman–Gallwey score	*Bifidobacterium*	87	−0.283	0.008	0.023	Significant
Menstrual irregularity/ovulatory dysfunction	*Lactobacillus*	87	0.125	0.247	0.557	Not significant
Menstrual irregularity/ovulatory dysfunction	*Prevotella*	87	−0.015	0.892	0.937	Not significant
Menstrual irregularity/ovulatory dysfunction	*Bifidobacterium*	87	0.103	0.342	0.615	Not significant
PCOM on TVS	*Lactobacillus*	87	0.009	0.937	0.937	Not significant
PCOM on TVS	*Prevotella*	87	−0.302	0.005	0.020	Significant
PCOM on TVS	*Bifidobacterium*	87	0.019	0.860	0.937	Not significant

Correlation analysis was performed using Spearman rank correlation. Menstrual irregularity/ovulatory dysfunction and PCOM on TVS were coded as binary variables. Lower Cq values indicate higher target DNA signal; therefore, the direction of rho should be interpreted in relation to Cq, not absolute bacterial abundance.

**Table 5:** qPCR standard-curve quality-control summary derived from the supplied instrument-export spreadsheets.

QC Item	*Lactobacillus*	*Prevotella*	*Bifidobacterium*
Standard concentration range	1.5 × 10^8^ to 1.2 × 10^9^ nominal units	1.5 × 10^8^ to 1.2 × 10^9^ nominal units	1.5 × 10^8^ to 1.2 × 10^9^ nominal units
Standard replicate structure	Duplicate levels	Duplicate levels; one 3.0 × 10^8^ replicate was discordant	Duplicate levels
Slope	−5.438	−5.714 with all standards; −4.021 after sensitivity exclusion of the discordant replicate	+1.766
Y-intercept	71.947	73.823 with all standards; 57.941 after sensitivity exclusion	20.386
R2	0.966	0.249 with all standards; 0.938 after sensitivity exclusion	0.058
Calculated efficiency	52.7%	49.6% with all standards; 77.3% after sensitivity exclusion	Not interpretable because the slope was positive
Duplicate standard Cq spread	0.056 to 0.318 Cq	0.283 to 10.925 Cq, driven by the discordant standard replicate	0.306 to 2.444 Cq
Negative-control status	NC amplified late at Cq 35.76	Negative-control well amplified late at Cq 36.42	No negative-control well was identifiable in the supplied export
Interpretive decision	Cq signal retained for exploratory analysis only; absolute quantity output not treated as validated	Cq signal retained as exploratory molecular signal only; no absolute load, assay validation, or clinical cutoff claim	Secondary exploratory finding only; quantitative or biological interpretation requires technical revalidation

The standard range refers to nominal standard concentrations in the instrument-export files. Efficiency values outside common qPCR performance expectations, late negative-control amplification, absent extraction blanks, absent LOD/LOQ experiments, and absent inter-run reproducibility data prevented absolute CFU/mL or copy-number interpretation. Therefore, the inferential analysis remained restricted to Cq-based exploratory signals.

## Data Availability

The data supporting the findings of this study are available from the corresponding author upon reasonable request. The revised analysis incorporated available qPCR instrument-export spreadsheets for *Lactobacillus*, *Prevotella*, and *Bifidobacterium* standard-curve QC. These exports should be retained with the analytical dataset. Raw fluorescence amplification plots, melt-peak outputs, negative-control details, extraction blank results, replicate-level Cq files, LOD/LOQ experiments, normalization-control data, and clinical covariate files should also be retained for editorial or reviewer inspection where available.

## Supplementary Materials

The following supporting information can be downloaded at: https://www.mdpi.com/article/10.3390/diagnostics16132041/s1, Table S1. Complete pairwise post-hoc comparisons of salivary microbial Cq signals across PCOS phenotypes and controls; Table S2. Exploratory ROC analysis of *Prevotella* for selected phenotype and control comparisons; Table S3. Age-adjusted regression analysis of *Prevotella* signal among women with PCOS; Table S4. Primer sequences and qPCR conditions for salivary microbiota quantification; Table S5. qPCR standard-curve quality-control summary from available instrument files.
