# Supplementary material for: Salivary Prevotella qPCR Signal as an Exploratory Non-Invasive Adjunct for Rotterdam Phenotype Stratification in Women with Polycystic Ovary Syndrome: A Proof-of-Concept Cross-Sectional Study
Source: Diagnostics (Basel). 2026 Jun 30;16(13):2041. doi: 10.3390/diagnostics16132041 (PMC13361299; doi:10.3390/diagnostics16132041)
Supplement: Supplementary file 1 [file diagnostics-16-02041-s001.zip › diagnostics-4364295-supplementary.pdf]

Supplementary Tables

Supplementary Table S1. Complete pairwise post-hoc comparisons of salivary microbial Cq signals across PCOS phenotypes and controls.

Microbiota	Group 1	Group 2	Raw p-value	Bonferroni-adjusted p-value	Significant after correction
Prevotella	Phenotype A	Phenotype B	0.370	1.000	No
Prevotella	Phenotype A	Phenotype C	0.128	1.000	No
Prevotella	Phenotype A	Phenotype D	<0.001	0.010	Yes
Prevotella	Phenotype A	Control	<0.001	0.010	Yes
Prevotella	Phenotype B	Phenotype C	0.039	0.390	No
Prevotella	Phenotype B	Phenotype D	<0.001	0.010	Yes
Prevotella	Phenotype B	Control	<0.001	0.010	Yes
Prevotella	Phenotype C	Phenotype D	0.002	0.020	Yes
Prevotella	Phenotype C	Control	0.004	0.040	Yes
Prevotella	Phenotype D	Control	0.249	1.000	No
Bifidobacterium	Phenotype A	Phenotype B	0.139	1.000	No
Bifidobacterium	Phenotype A	Phenotype C	0.388	1.000	No
Bifidobacterium	Phenotype A	Phenotype D	0.012	0.120	No
Bifidobacterium	Phenotype A	Control	<0.001	0.010	Yes
Bifidobacterium	Phenotype B	Phenotype C	0.388	1.000	No
Bifidobacterium	Phenotype B	Phenotype D	0.283	1.000	No
Bifidobacterium	Phenotype B	Control	0.018	0.180	No
Bifidobacterium	Phenotype C	Phenotype D	0.019	0.190	No
Bifidobacterium	Phenotype C	Control	<0.001	0.010	Yes
Bifidobacterium	Phenotype D	Control	0.199	1.000	No

Pairwise comparisons were performed following the Kruskal-Wallis test. Bonferroni-adjusted p-values are shown. Lower Cq values indicate higher target DNA signal.

Supplementary Table S2. Exploratory ROC analysis of *Prevotella* for selected phenotype and control comparisons.

Comparison	Microbiota	Positive group	n positive	n negative	Raw AUC original direction	Direction-adjusted AUC	AUC 95% CI lower	AUC 95% CI upper	Exploratory threshold rule	Sensitivity	Specificity	Raw p-value	Direction note
Phenotype A vs Phenotype D	Prevotella	Phenotype D	23	23	0,223	0,777	0,622	0,916	Prevotella qPCR quantity ≤ 8.80479e+06	0,783	0,826	1,338E-03	Lower Prevotella values indicate the positive group
Phenotype B vs Control	Prevotella	Control	23	19	0,144	0,856	0,723	0,963	Prevotella qPCR quantity ≤ 3.86165e+07	0,913	0,737	8,968E-05	Lower Prevotella values indicate the positive group
Phenotype C vs Control	Prevotella	Control	23	22	0,237	0,763	0,609	0,897	Prevotella qPCR quantity ≤ 5.1984e+06	0,652	0,818	2,625E-03	Lower Prevotella values indicate the positive group
Phenotype D vs hyperandrogenic phenotypes	Prevotella	Phenotype D	23	64	0,210	0,790	0,653	0,908	Prevotella qPCR quantity ≤ 1.92066e+06	0,696	0,875	4,043E-05	Lower Prevotella values indicate the positive group

ROC results are exploratory only and should not be used as clinical diagnostic cut-offs. Thresholds refer to standard-curve-derived software quantity outputs retained from the analysis file, not validated CFU/mL bacterial load.

Supplementary Table S3. Age-adjusted regression analysis of *Prevotella* signal among women with PCOS.

Predictor	Coefficient	Standard error	95% CI lower	95% CI upper	p-value	Interpretation
Intercept	7,123	0,794	5,543	8,704	1,483E-13	Significant
Age	0,013	0,027	-0,040	0,066	6,367E-01	Not significant
Phenotype B vs A	-0,007	0,367	-0,738	0,723	9,838E-01	Not significant
Phenotype C vs A	-0,155	0,341	-0,834	0,524	6,512E-01	Not significant
Phenotype D vs A	-1,435	0,350	-2,132	-0,738	1,029E-04	Significant

Regression was retained as an exploratory supportive analysis. Coefficients should be interpreted in relation to the qPCR scale used in the original analysis file.

Supplementary Table S4. Primer sequences and qPCR conditions for salivary microbiota quantification.

Genus	Forward primer sequence (5′-3′)	Reverse primer sequence (5′-3′)	qPCR platform/condition	Revised reporting note
Lactobacillus	AGCAGTAGGGAATCTTCCA	CACCGCTACACATGGAG	MyGo Mini S; SYBR Green; annealing 60 deg C	Reported as Cq-based microbial signal; no GAPDH-normalized Delta Delta Ct analysis used.
Prevotella	TCGCGTCYGGTGTGAAAG	GGTGTTCCCGATATCTACA	MyGo Mini S; SYBR Green; annealing 60 deg C	Reported as Cq-based microbial signal; no GAPDH-normalized Delta Delta Ct analysis used.
Bifidobacterium	GAGGCGATGGTCTGGAAGTT	CCACATCGCCGAGAAGATTC	MyGo Mini S; SYBR Green; annealing 50 deg C	Reported as Cq-based microbial signal; no GAPDH-normalized Delta Delta Ct analysis used.

The manuscript reports Cq-based microbial signals. Lower Cq values indicate higher target DNA signal. Primer validation and standard-curve performance should be rechecked before claims of absolute bacterial load are made.

Supplementary Table S5. qPCR standard-curve quality-control summary from available instrument files.

Target	Instrument file used for revised QC	Standard range	Annealing temperature	Slope	Intercept	R²	Estimated efficiency	Negative control status	Manuscript implication
Prevotella	2025-04-14 standard-sample run	1.5 x 10^8 to 1.2 x 10^9	60 deg C	-5.274	63.372	0.9255	54.7%	NC amplified at Cq 36.42	Use as exploratory Cq signal; avoid absolute CFU/mL claim
Lactobacillus	2025-04-09 standard-sample run	1.5 x 10^8 to 1.2 x 10^9	60 deg C	-5.438	71.947	0.9662	52.7%	NC amplified at Cq 35.76	Use cautiously; no absolute load claim
Bifidobacterium	2025-04-09 standard-sample run	1.5 x 10^8 to 1.2 x 10^9	50 deg C	-2.308	56.149	0.8815	171.1%	NC not clearly documented in available file	Secondary finding; technical revalidation recommended
Bifidobacterium	2025-03-14 standard-sample run	1.5 x 10^8 to 1.2 x 10^9	50 deg C	1.766	20.386	0.0583	Invalid	NC not clearly documented	Do not use for quantitative interpretation

Efficiency was estimated from slope using E = 10^(-1/slope) - 1, where applicable. These QC findings justify revising the manuscript from absolute/relative abundance language to cautious Cq-signal language.
